# The quest for blood pressure markers in photoplethysmography and its applications in digital health

**DOI:** 10.3389/fdgth.2025.1518322

**Published:** 2025-04-30

**Authors:** Josep Sola, Andreu Arderiu, Tiago P. Almeida, Sibylle Fallet, Sasan Yazdani, Serj Haddad, David Perruchoud, Olivier Grossenbacher, Jay Shah

**Affiliations:** Aktiia SA, Neuchâtel, Switzerland

**Keywords:** photoplethysmography, cuffless blood pressure, hypertension, optical blood pressure monitor, continual blood pressure monitoring

## Abstract

**Introduction:**

Photoplethysmography (PPG) sensors, capturing optical signals from arterial pulses, are debated for their potential in blood pressure (BP) measurement. This study employed the largest dataset to date of paired PPG and cuff BP readings to explore PPG signals for BP estimation.

**Methods:**

32,152 European residents (age 55.9% ± 11.8, 24% female, BMI 27.7 ± 4.6) voluntarily acquired and used a cuffless BP monitor (Aktiia SA, Switzerland) between March/2,021-March/2023. Systolic and diastolic BP (SBP, DBP) from an upper arm oscillometric cuff were collected simultaneously with wrist PPG (668,080 paired measurements). Six different machine learning models were developed to predict BP using cuff BP readings as reference (75%|15%|15% training|validation|testing): four baseline models [heart rate (HR), Age, Demography (DEM: Age + Gender + BMI), DEM + HR], and two models relying on the analysis of the PPG waveforms (PPG, PPG + DEM). Performance of each model was evaluated on the 4,823 subjects from the testing set using as metrics the Pearson's correlation (r) when comparing the estimated and the reference BP values, and the area under the receiver operating characteristic (AUROC) curves, and true positive and true negative rates (TPR, TNR) for the detection of high BP (reference SBP ≥ 140 or DBP ≥ 90 mmHg, applying a ± 8 mmHg exclusion zone to account for cuff measurement uncertainty).

**Results:**

Baseline models showed low correlation with cuff data and poor high BP detection (*r* < 0.35; AUROC < 0.65, TPR < 0.65, TNR < 0.58). PPG-based models excelled in correlating with cuff BP (SBP: *r* = 0.53 for PPG, *r* = 0.63 for PPG + DEM; DBP: *r* = 0.58 for PPG, *r* = 0.67 for PPG + DEM) and high BP detection (SBP: AUROC = 0.84, TPR = TNR = 0.75; DBP: AUROC = 0.89, TPR = TNR = 0.81 for PPG; SBP: AUROC = 0.89, TPR = TNR = 0.80; DBP: AUROC = 0.93, TPR = TNR = 0.86 for PPG + DEM).

**Discussion:**

This study demonstrated that PPG signals contain reliable markers of BP, and that BP values can be estimated using only markers found within PPG's optical pulsatility signals, outperforming models based solely on demographic data. These findings hold the potential to radically transform hypertension screening and global healthcare delivery, paving the way for innovative approaches in patient diagnosis, monitoring and treatment methodologies.

## Introduction

Photoplethysmography (PPG) has become a prevalent optical technique for monitoring both patients in clinical settings and individuals in their daily lives. PPG data have been successfully used in measuring hemodynamic and cardiovascular parameters, such as blood oxygen saturation and heart rate (HR), now commonly used and widely accepted in healthcare (1, 2). There is growing interest in utilizing PPG for novel approaches to blood pressure (BP) measurements. However, recent solutions rely on complementary data, like electrocardiograms (ECG) or phonocardiograms (PCG) (3–9), or require regular calibration with standard cuff devices (10–13). These practical limitations hinder the widespread adoption of cuffless BP technology. Additionally, there is intense debate on whether PPG signals truly contain markers that can serve as indirect surrogates for BP measurements (14). Moreover, the feasibility of using PPG data alone to detect high BP or monitor hypertension without any *a priori* calibration—or complementary data—has been demonstrated in few works and need further investigation (15–22). Finally, a crucial aspect missing from this discussion is a direct comparison between BP estimates derived purely from physiological data—*e.g.*, age, height, and weight—against those derived from PPG inputs (21). Successfully addressing these challenges could pave the way to a meaningful and scalable change in global BP and hypertension screening, diagnosis and management.

Recent advancements in machine learning (ML), particularly in deep learning techniques, have catalysed the development of novel models that, once trained on large-scale datasets, excel at extracting features and recognizing patterns from intricate data, outperforming classical methods (23). In this study, we evaluated the potential of using markers embedded in PPG signals to directly estimate BP values, eliminating the need for external calibration or additional data sources. Using the largest dataset to date of PPG and cuff BP readings collected simultaneously, we developed ML models incorporating diverse physiological inputs—including age, gender, and PPG—to predict BP values. The effectiveness of these models was assessed by comparing the resulting BP estimation against reference cuff BP measurements.

## Methods

### Study population

This retrospective study included data from 32,152 European residents (Table 1, 55.9 ± 11.8 years old, 24% female, BMI 27.7 ± 4.6 kg/m²). All users voluntarily purchased and wore a validated, CE-marked, over-the-counter cuffless wrist BP monitor (Aktiia SA, Neuchâtel, Switzerland) between March/2021 and March/2023 were included in the investigation (12, 13). All methods were carried out in accordance with relevant guidelines and regulations. The present work used retrospective anonymized data collected on users of a commercial CE-marked BP monitor with no associated experimental protocol, therefore no evaluation from an ethic committee was required. Informed consent was obtained from all subjects through the commercial agreement of usage for the Aktiia monitor. Prior diagnoses of hypertension and medications taken were unknown. During installation and setup of the Aktiia smartphone application, the user is asked to provide personal information, including age, gender, height and weight.

**Table 1:** Characteristics of the users included in this study.

	Training			Testing	All data
	Training	Validation	All training		
Users’ characteristics					
Count	22,506	4,823	27,329	4,823	32,152
Vs. all data, %	70.0%	15.0%	85.0%	15.0%	100.0%
Age, years	55.8 ± 11.9	56.1 ± 11.6	55.9 ± 11.9	55.8 ± 11.7	55.9 ± 11.8
Height, cm	176.0 ± 9.0	176.3 ± 8.9	176.1 ± 9.0	176.1 ± 8.8	176.1 ± 9.0
Weight, kg	86.1 ± 16.4	86.1 ± 16.4	86.1 ± 16.4	86.3 ± 16.2	86.1 ± 16.4
BMI, kg/m²	27.7 ± 4.6	27.6 ± 4.6	27.7 ± 4.6	27.8 ± 4.6	27.7 ± 4.6
Gender, female %	25%	24%	24%	24%	24%
Simultaneous PPG and cuff recordings					
Count	468,698	100,010	568,708	99,372	668,080
Vs. all data, %	70.2%	15.0%	85.1%	14.9%	100.0%
p user, median [IQR]	11 [5–26]	11 [5–26]	11 [5–26]	11 [4–26]	11 [5–26]
Cuff-based BP profile during first day					
Systolic BP (mmHg)	134.6 ± 16.9	134.6 ± 16.9	134.6 ± 16.9	134.5 ± 17.0	134.5 ± 16.9
Diastolic BP (mmHg)	83.4 ± 11.5	83.6 ± 11.5	83.5 ± 11.5	83.3 ± 11.5	83.4 ± 11.5

All data represented as mean ± SD, unless stated otherwise. BMI, body mass index; PPG, photoplethysmography; BP, blood pressure.

### Data collection

The Aktiia monitor is comprised of a bracelet that collects green reflective PPG signals from the wrist, and an oscillometric brachial cuff used for initializations (*i.e.*, calibrations) performed at least once a month (24). Initialization consists of cuff BP measurements performed by the Aktiia upper-arm cuff collected simultaneously to 30 s of PPG data performed by the Aktiia bracelet. Therefore, during initializations, each PPG segment is associated with a cuff BP reading. During this process, users are required to sit still while measurements are performed. The procedure is fully automated and controlled by the Aktiia smartphone application, with a series of signal quality tests (25). In case the signals collected during initialization fail quality tests, the measurements are discarded, and the user is requested to repeat the initialization. The data from the bracelet and cuff are transferred via Bluetooth to the smartphone application, and forwarded to Aktiia's cloud server, where they are stored (26).

In the present work, 668,080 cuff SBP and DBP readings [approximately 11 (5–26) measurements/user, median (IQR)] recorded simultaneously with PPG signals during initializations performed from March 2021 to March 2023 were included in the analysis (Table 1).

### ML models for BP estimation

**Figure 1:**
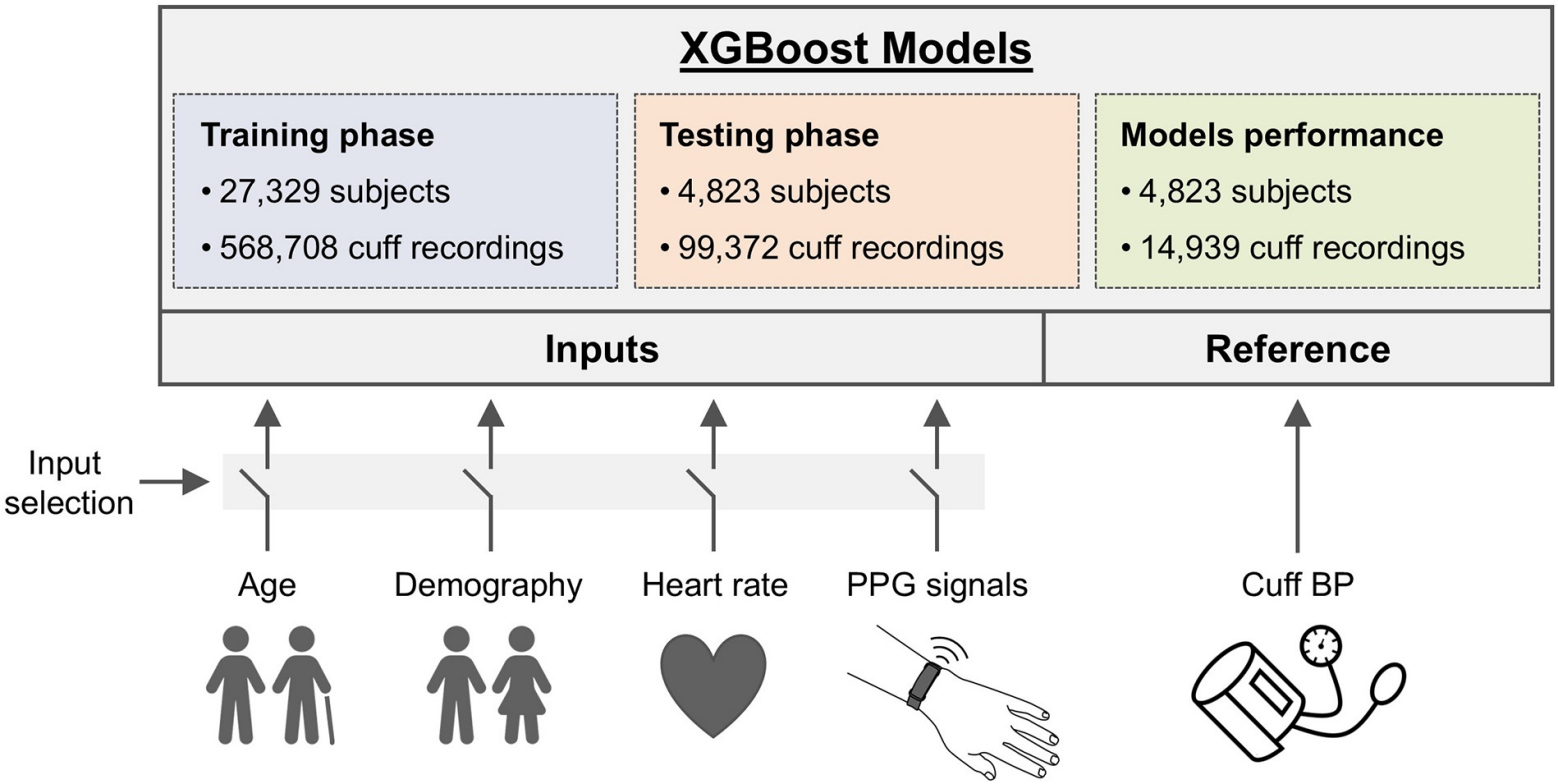
Framework of the study. A total of 668,080 systolic and diastolic BP (SBP, DBP) from an upper arm oscillometric cuff were collected simultaneously with wrist PPG from 32,152 European residents. Six XGBoost models with different input setups were created to predict BP, using cuff readings for training. Four baseline models incorporated inputs like heart rate (HR), age, and Demography (DEM) data (age, gender, BMI), either individually or combined, while two PPG models utilized PPG signals, with and without Demography data. Model training was performed on 85% of users (27,329 users with 568,708 cuff recordings), and testing was conducted on the remaining 15% of users (4,823 individuals with 99,372 cuff readings). The performance analyses were conducted using only readings from the first day of each user's monitoring period on the testing dataset (14,939 cuff readings).

Six ML regression models were developed to estimate SBP and DBP, utilizing cuff BP readings as the training reference. A framework of the study is illustrated in Figure 1 and detailed in the Supplementary Materials. The data distribution was set at 85% for training (*n* = 27,329 users), split into 70% for actual training (*n* = 22,506) and 15% for validation (*n* = 4,823), as detailed in Table 1. The remaining 15% (*n* = 4,823) were used for testing. This resulted in a dataset of 468,698 cuff readings for training, 100,010 for validation, and 99,372 for testing. To ensure data integrity, users used in the testing phase were not used during training and validation stages, assuring that only new and independent data from distinct users were employed for model testing.

In this study, the XGBoost algorithm was the ML method chosen for all models (27). In each case, different XGBoost models were developed with different input setups aimed to predict BP using cuff BP readings as reference. XGBoost excels at identifying nonlinear relationships and handling large datasets efficiently. It is a convenient tool for constructing models based on tabular data like the one present in this study, allowing to assess how physiological data (demographic or PPG) impacts the accuracy of BP estimation. Details about the parameter configuration used can be found in Supplementary Materials.

### Baseline models

Four baseline XGBoost models were created using a variety of physiological data as predictors, excluding PPG, to assess the influence of such data on BP estimation. These models serve as baseline benchmarks that enable assessing whether incorporating PPG signals enhances BP estimation accuracy beyond what is achievable with models relying solely on physiological data. Predictors for these models included cuff HR, Age, a composite termed ‘Demography’ (DEM, encompassing age, gender, height and weight), and a combination of DEM + HR. Except for HR, physiological data were treated as static for each user throughout the study. The physiological data was adjusted to the last update provided by the users at the moment of data collection.

### PPG-based models

Two XGBoost models were specifically designed to use PPG signals as predictors: one using only PPG data and another combining PPG with DEM data. For these models, each 30-s PPG segment corresponded with its respective cuff BP reading in both the training and testing datasets. Similar to the baseline models, DEM data were considered fixed for each user and replicated across all cuff BP readings for consistency in analysis, considering the last update provided by the users at the moment of data collection. As the XGBoost model is best suited to work with tabular data, PPG signals underwent a pre-processing step to eliminate noise, and key features were extracted and presented as inputs to the XGBoost model to predict cuff BP values (28–31). Additionally, for the PPG + DEM model, demographic data is concatenated with these PPG features to improve the model's predictive accuracy.

### Statistical analysis

The effectiveness of the models was evaluated using data from the testing phase. The estimated SBP and DBP by each model were compared to actual cuff BP readings through Pearson's correlation coefficient (r). Correlation analysed across different demographic groups was included in the Supplementary Materials: gender (male and female), age categories (adult and elderly, the latter defined as age over 65 years), and BMI classifications (normal weight for BMI < 25, overweight for 25 ≤ BMI < 30, and obese for BMI ≥ 30). The samples were bootstrapped (10,000 replications) to estimate the 95% confidence interval (CI) of the mean correlation.

Receiver operating characteristic (ROC) curves were created to test the performance of the model to correctly distinguish high BP. High BP was investigated separately for SBP and DBP. Different criteria for high BP were investigated for both cuff SBP (thresholds varying from ≥120 mmHg to ≥180 mmHg) and DBP (thresholds varying from ≥80 mmHg to ≥110 mmHg). An exclusion zone of ±8 mmHg was applied around the detection thresholds (*i.e.*, reference BP readings within the exclusion zone were not accounted in the performance calculations) to account for cuff measurement uncertainty (32). The true positive rate (TPR) and true negative rate (TNR) were determined at the optimal operating point on each ROC curve—defined as the closest point to the graph's top left corner. The area under the ROC curve (AUC) was calculated to further assess high BP estimation performance. The performances without the ±8 mmHg exclusion zone are provided in the Supplementary Materials.

The analyses for the testing included only readings from the first day of each user's monitoring period (14,939 cuff readings). *P*-values less than 0.05 were considered statistically significant.

## Results

There were approximately 11 readings per user across the entire dataset [median (IQR), 11 (5–26)]. The training and validation dataset had each 11 [5–26] readings per user. The entire testing dataset had 11 [4–26] readings per user, while the first day of the testing dataset (used on the performance analyses) had 3 [2–3] readings per user. The overall average SBP was 134.5 ± 16.9 mmHg and DBP was 83.4 ± 11.5 mmHg. These findings are summarized in Table 1. The distribution of study duration per participant can be found in the Supplementary Materials.

### Correlation of BP estimates: models vs. cuff measurements

**Figure 2:**
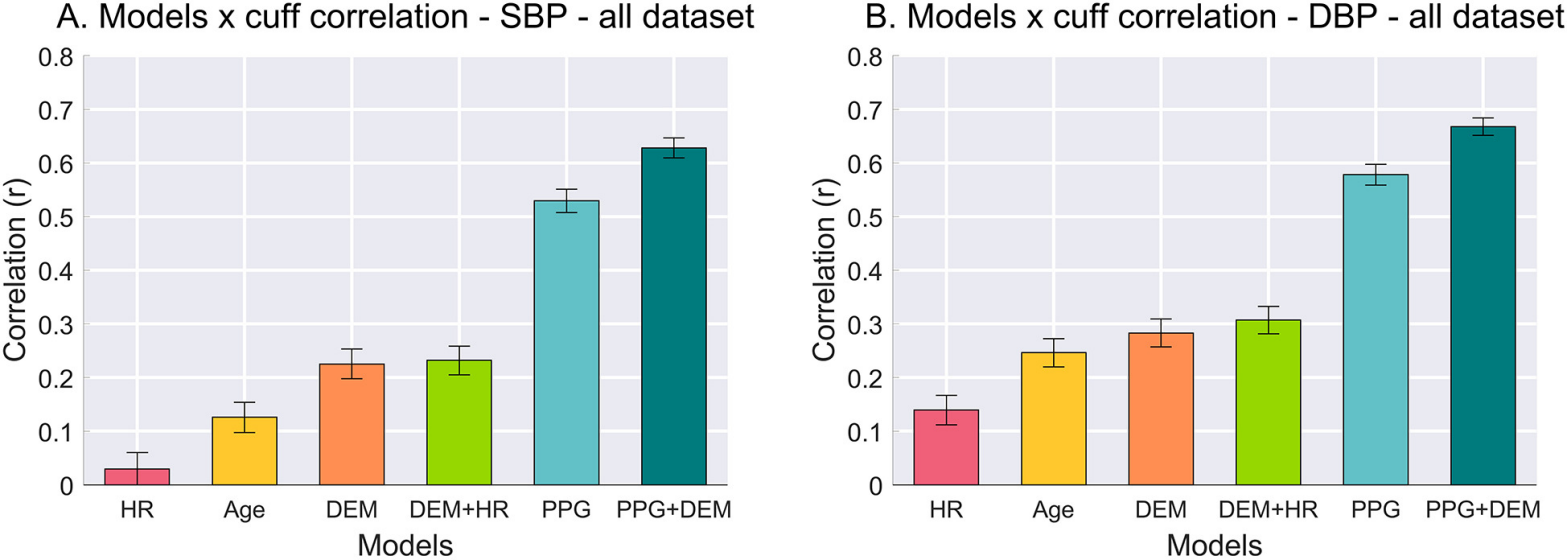
Pearson's correlation between BP estimates from each model vs. cuff BP values. **(A)** Correlation for SBP. **(B)** Correlation for DBP. Error bars represent 95% confidence interval (CI) of the mean correlation calculated from bootstrapping the samples (10,000 replications).

BPs estimated with baseline models showed poor correlation vs. the reference cuff BP, as illustrated in Figures 2A,B. Specifically, for SBP, the HR model resulted in correlation of *r* = 0.03, the Age model *r* = 0.13, and both DEM and DEM + HR models *r* = 0.23 (*P* < 0.0001 in all cases). Similarly, DBP estimation correlations were *r* = 0.14 for the HR model, *r* = 0.25 for the Age model, *r* = 0.28 for the DEM model, and *r* = 0.31 for the DEM + HR model (*P* < 0.0001 in all cases).

In contrast, the correlation between PPG-based models vs. the reference cuff BP outperformed all baselines. The PPG model achieved an SBP correlation of *r* = 0.53, and the PPG + DEM model reached *r* = 0.63 (*P* < 0.0001 for both). For DBP, the correlations were even higher: *r* = 0.58 for the PPG model and *r* = 0.67 for the PPG + DEM model (*P* < 0.0001 for both).

### High BP estimation

**Figure 3:**
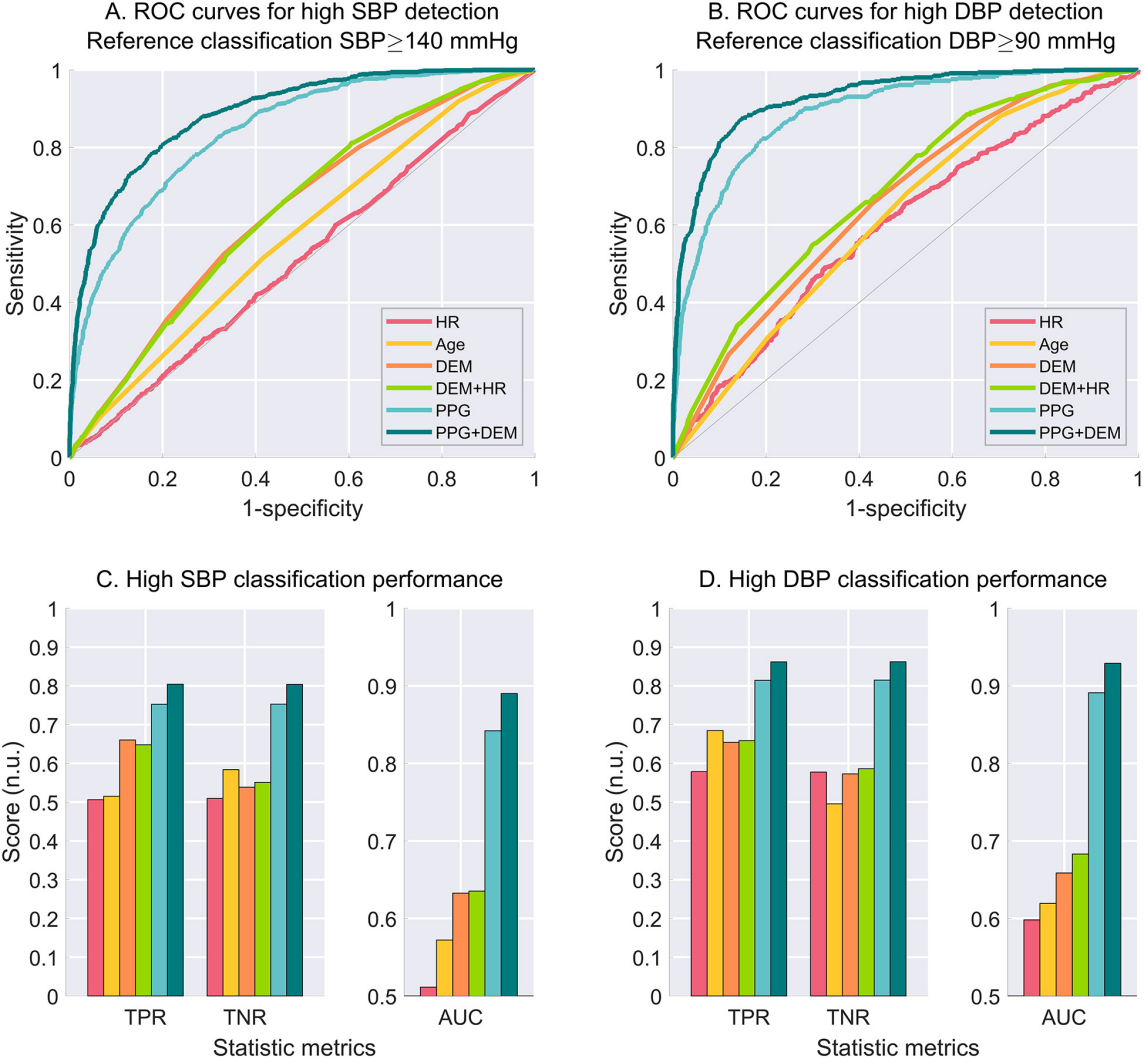
Performance for high BP estimation for each model considering the criterion for high BP set at cuff SBP ≥ 140 mmHg (left-hand side), and cuff DBP ≥ 90 mmHg (right-hand side). **(A)** ROC curves for high SBP estimation. **(B)** ROC curves for high DBP estimation. C. Estimation performance metrics as true positive rate (TPR), true negative rate (TNR) and area under the curve (AUC) for high SBP. **(C)** Estimation performance metrics for high DBP. An exclusion zone of ±8 mmHg was adopted for the creation of the ROC curves to account for cuff measurement uncertainty. N.u., no unit.

A visual representation of the ROC curves and the metrics used to assess the performance of high BP estimation for each model is shown in Figure 3, considering only one criterion for high SBP (≥140 mmHg) and DBP (≥90 mmHg), with a ± 8 mmHg exclusion zone adopted to account for cuff measurement uncertainty.

The estimation performance for different criteria for high SBP and high DBP are detailed on Tables 2,3, respectively. PPG-based models demonstrated superior BP estimation capabilities compared to baseline models for all metrics and all criteria, particularly when DEM data were incorporated.

**Table 2:** Estimation performance of high SBP for each model considering different criteria of high SBP.

Metric	Model	Threshold for high SBP, mmHg				
		≥120	≥130	≥140	≥160	≥180
TPR						
	HR	0.491	0.514	**0**.**506**	0.530	0.500
	Age	0.472	0.500	**0**.**515**	0.536	0.654
	DEM	0.595	0.624	**0**.**660**	0.569	0.615
	DEM + HR	0.598	0.614	**0**.**648**	0.558	0.577
	PPG	0.716	0.738	**0**.**753**	0.801	0.769
	PPG + DEM	0.808	0.801	**0**.**804**	0.812	0.808
TNR						
	HR	0.494	0.505	**0**.**510**	0.532	0.514
	Age	0.555	0.588	**0**.**584**	0.561	0.551
	DEM	0.661	0.597	**0**.**538**	0.623	0.608
	DEM + HR	0.658	0.612	**0**.**551**	0.618	0.603
	PPG	0.716	0.738	**0**.**753**	0.801	0.780
	PPG + DEM	0.807	0.801	**0**.**804**	0.813	0.814
AUC						
	HR	0.492	0.511	**0**.**511**	0.549	0.515
	Age	0.545	0.567	**0**.**572**	0.574	0.643
	DEM	0.678	0.653	**0**.**633**	0.627	0.626
	DEM + HR	0.677	0.658	**0**.**635**	0.630	0.623
	PPG	0.801	0.815	**0**.**842**	0.885	0.894
	PPG + DEM	0.887	0.886	**0**.**890**	0.903	0.891

True positive rate (TPR) and true negative rate (TNR) were calculated considering the optimum operating point from the ROC curves of each model, while the area under the curves (AUC) were extracted directly from the ROC curves. The bold values referring to threshold SBP ≥ 140 mmHg are illustrated in Figure 3. A ±8 mmHg exclusion zone was adopted to account for cuff measurement uncertainty.

**Table 3:** Estimation performance of high DBP for each model considering different criteria of high DBP.

Metric	Model	Threshold for high DBP, mmHg				
		≥80	≥85	≥90	≥100	≥110
TPR						
	HR	0.586	0.579	**0**.**579**	0.588	0.697
	Age	0.674	0.692	**0**.**685**	0.695	0.727
	DEM	0.644	0.651	**0**.**654**	0.626	0.576
	DEM + HR	0.672	0.663	**0**.**659**	0.542	0.515
	PPG	0.812	0.804	**0**.**814**	0.824	0.818
	PPG + DEM	0.854	0.857	**0**.**862**	0.863	0.848
TNR						
	HR	0.584	0.579	**0**.**578**	0.588	0.697
	Age	0.581	0.539	**0**.**495**	0.443	0.422
	DEM	0.692	0.629	**0**.**573**	0.506	0.483
	DEM + HR	0.690	0.649	**0**.**586**	0.624	0.599
	PPG	0.812	0.804	**0**.**815**	0.824	0.818
	PPG + DEM	0.854	0.856	**0**.**862**	0.863	0.848
AUC						
	HR	0.605	0.595	**0**.**598**	0.622	0.727
	Age	0.673	0.653	**0**.**619**	0.585	0.594
	DEM	0.723	0.692	**0**.**659**	0.616	0.563
	DEM + HR	0.743	0.714	**0**.**683**	0.641	0.628
	PPG	0.887	0.887	**0**.**891**	0.916	0.922
	PPG + DEM	0.932	0.932	**0**.**929**	0.942	0.939

True positive rate (TPR) and true negative rate (TNR) were calculated considering the optimum operating point from the ROC curves of each model, while the area under the curves (AUC) were extracted directly from the ROC curves. The bold values referring to threshold DBP ≥ 90 mmHg are illustrated in Figure 3. A ±8 mmHg exclusion zone was adopted to account for cuff measurement uncertainty.

## Discussion

In the present work, we provide direct evidence that green reflective PPG signals contain markers that allow for BP estimation without the need for external calibration nor additional inputs, such as ECG or PCG. To the best of our knowledge, this is the first large-scale study to present direct comparison of BP estimates obtained solely from physiological data—*e.g.*, age and gender—against those obtained with PPG inputs. Our findings reveal that, using the same model topology based on XGBoost with different input settings, the intrinsic characteristics of PPG signals enable effective BP estimation, far exceeding the predictive value of basic physiological descriptors. These physiological descriptors, when isolated, lack the depth needed for robust BP estimation. Specifically, the fiducial points in the PPG waveform—that can be extracted through the proprietary signal processing algorithm—carry BP-related features. These features were used as input variables of the two PPG-based models that resulted in effective BP estimation. Conversely, the basic physiological descriptors (*i.e.*, variables such as age, gender, height, weight, and HR) are insufficient for accurate BP estimation when used isolated. The models using PPG outperformed these descriptors, and performance improves further when combined with them. The results reported reflect model performance using off-the-shelf XGBoost technologies and do not represent the performance of any medical device by the study sponsor.

### From markers within PPG signal to BP estimation

The PPG signal is composed of reflective waves from the central arterial system that can be detected at peripheral arteries by optical sensors—*i.e.*, the microvascular bed in the upper layers of the skin (33). They originate from the aorta shortly after mechanical contraction of the left ventricle as the arterial wave propagates through the central arterial tree. Reflections of the waves occur at points of arterial division or where branches of different diameters merge, such as at the juncture of the subclavian artery and thoracic aorta or the iliac bifurcation. These sites create an impedance mismatch, causing the reflections that, when superimposed on the primary ventricular ejection pulse, form the characteristic waveform seen in PPG signals collected at peripheral sites (33).

The PPG waveform carries thus distinct markers influenced by hemodynamic and cardiovascular factors, particularly arterial stiffness, indicating the PPG signals contain markers that could provide insights into arterial circulation, thereby informing about BP levels (34–42).

Within the PPG waveform, fiducial points—such as the systolic peak, dicrotic notch, and diastolic peak—represent relevant physiological events in the cardiac cycle. These features are shaped by vascular resistance, pulse wave velocity, and arterial elasticity, all of which directly correlate with BP dynamics (33). Variations in the timing, amplitude, and morphology of these waveform components can indicate underlying hemodynamic changes, offering valuable insights into arterial circulation. Machine learning models can leverage these patterns to predict SBP and DBP with greater precision, provided that they have been trained with large and representative enough datasets. Furthermore, derived indices such as the augmentation index and reflection index, embedded within the PPG signal, provide additional cardiovascular metrics relevant to BP estimation.

This physiological foundation underscores the potential of PPG for BP estimation without the need of a cuff—it encapsulates both arterial mechanics and the dynamic interplay between the heart and vascular system, making it a promising tool for non-invasive BP monitoring.

Our results support the conclusion that PPG signals harbour markers that are effective for estimating BP. Furthermore, our analysis demonstrated that BP estimates derived from PPG signals successfully discriminate between users with high and normal BP (considering a ± 8 mmHg exclusion zone to account for cuff measurement uncertainty; results without exclusion zone can be found in the Supplementary Materials, and corroborate the results for effective high and normal BP detection). In contrast, estimates based only on HR, age or on detailed demographic descriptors, including BMI and gender, were ineffective in this discrimination. Therefore, caution is needed when employing BP estimation methods that consider only combinations of demographic data, without including information derived from PPG data.

### Cuffless BP technologies

Current cuffless BP technologies effectively employ PPG to identify BP fluctuations around an offset that is defined during initialization usually conducted with cuff-based devices (10–13), or combine PPG with external references (*e.g.*, ECG, PCG) to describe the velocity of pressure pulse propagation (3–9). Either way, the need of cuff for initializations or external references restricts the wider acceptance and adoption of cuffless BP monitors by both healthcare providers and the general public.

Recent works have implemented PPG-based solutions without the need of initializations or external references (15–22). While previous studies have significantly advanced our understanding, many of them suffer from common caveats, including using curated datasets with specific characteristics or data distribution, small datasets, train/test data leakage and misleading performance metrics (3–13, 15–22). In the present work, we harness the largest dataset to date of PPG signals collected with a commercialized cuffless monitor for estimating absolute BP values without relying on a calibration-dependent offset. Additionally, PPG-based models resulted in superior BP assessment capabilities compared to baseline models irrespective of the definition of high BP (and with and without a ± 8 mmHg exclusion zone to account for cuff measurement uncertainty). Importantly, the different thresholds adopted in the present study to characterize high BP reflect current guidelines for diagnosis of hypertension, demonstrating its potential for hypertension management programs.

This breakthrough paves the way for a calibration-free solution applicable across a wide range of wearables. Such an innovation offers the promise of continuous BP monitoring without the discomfort of cuffs or reliance on external references, fulfilling a longstanding aspiration in healthcare technology. Our demonstration that PPG signals can estimate BP without the need for cuff-based calibration sets the stage for a shift in global BP care. It supports large-scale, long-term continuous BP assessments and sets the stage for a transformational change of BP and HTN management.

### Deep learning frameworks for BP estimation

In this study, XGBoost was selected due to its robustness in handling structured data and its efficiency with tabular features in large datasets. XGBoost is an ensemble learning method based on gradient-boosted decision trees, which iteratively improves model performance by minimizing errors from previous iterations. It is particularly effective for tabular data, handling missing values, capturing non-linear relationships, and providing high predictive accuracy with relatively low computational cost (28–31). This choice aligns with the study's focus on physiological data combined with extracted PPG features, where structured variables and complex interactions are key. Importantly, XGBoost demonstrated improved BP estimation when incorporating PPG data compared to baseline models, effectively showcasing the added value of PPG signals in BP prediction.

While deep learning models such as Convolutional Neural Networks (CNNs) and Long Short-Term Memory (LSTM) may further improve BP estimation, this study aimed to assess whether PPG signals contain BP-related markers and if a model leveraging them could outperform those based on demographics or heart rate, rather than comparing deep learning architectures. This objective was successfully achieved using XGBoost.

Future work will explore deep learning models to investigate their potential for capturing more complex temporal and non-linear features from PPG data, which may further enhance BP estimation accuracy. CNNs are particularly effective at learning spatial hierarchies from data, making them suitable for extracting features from raw PPG waveforms. For instance, Cho *et al*. developed a calibration-free BP estimation model using a 3-layer CNN with time-series ECG and PPG signals, achieving root mean square errors of 5.80 mmHg for SBP and 2.78 mmHg for DBP (43). Similarly, Sun *et al*. predicted BP risk levels (normotensive, prehypertensive, and hypertensive) using a combination of CNNs with the Hilbert-Huang Transform, achieving F1 scores as high as 98.90% (44). LSTM networks excel in modeling temporal dependencies, capturing the sequential nature of physiological signals. Zhao *et al*. applied an LSTM model to predict SBP and DBP using raw PPG signals from an animal model (45). Additionally, Kamanditya *et al*. developed a BP prediction system that integrates CNN and LSTM layers to merge extracted features from PPG and ECG signals, achieving accuracies of 5.31 ± 7.25 mmHg for SBP and 3.30 ± 4.76 mmHg for DBP (46). A hybrid CNN-LSTM architecture could theoretically leverage both spatial and temporal dynamics for more comprehensive BP estimation.

### Limitations

This study allocated the data across the training, validation, and testing sets in proportions of 75%, 15%, and 15%, respectively. This approach resulted in 99,372 data points from 4,823 users for the testing phase. The dataset reflects a wide variety of user behaviours, retention rates, and compliance levels. To ensure consistency and reduce temporal variations in BP, only data from the first day of collection for each of the 4,823 users was included in the results. This limitation was applied only to the testing dataset, while the training and validation datasets utilized all available data. By focusing on the first day, bias from more compliant users who may have calibrated their devices more frequently was avoided (*e.g.*, some users may have only performed a single calibration, while others may have calibrated their Aktiia bracelet every week for 2 years). Additionally, this approach provided a standardized snapshot of BP profiles at a single point in time, although it did not account for temporal changes over longer periods. The first day was chosen for consistency, as subsequent use might alter behaviour (*e.g.*, receiving a diagnosis and medication treatment).

The demographic data, provided by the users, are susceptible to entry errors, including typos. Moreover, these parameters, such as weight, could vary over time due to changes like weight gain or loss. It is worth noting that users might not have consistently updated their profiles to reflect such changes. Nevertheless, we believe the data is representative of the population and was used to train the models adequately.

The present work adopted an exclusion zone of ±8 mmHg to account for cuff measurement uncertainty (32). Exclusion zones are a well-established and essential component of standard practice, especially in cuff-based BP studies (47–49). Incorporating an exclusion zone based on the known uncertainty of the reference device is a disseminated approach, which can be extended to ROC analysis for sensitivity and specificity when the reference measurements are subject to a known variability.

When the reference device has a known level of measurement uncertainty, certain samples may fall within a gray zone where the classification as true positive or false negative becomes arbitrary. Including these uncertain samples can introduce misclassification bias, distorting the ROC curve and potentially leading to incorrect interpretations of model performance. The exclusion zone helps mitigate this issue by reducing the impact of measurement noise, thereby enhancing the robustness and reliability of the performance metrics.

In this study, the ±8 mmHg exclusion zone was defined based on the known standard deviation associated with cuff-based BP measurements (32). This approach aligns with practices in medical device validation, where biological variability and instrument imprecision are acknowledged as factors that can influence diagnostic performance. Regulatory bodies such as the FDA and international standards such as ISO recognize the importance of accounting for variability and measurement uncertainty in process validation. The use of exclusion zones, particularly in the context of diagnostic accuracy studies, is well aligned with this practice (32, 50).

Moreover, it is important to emphasize that the exclusion zone was applied consistently across all models in the present work, including both baseline and PPG-based models. This ensures that the influence of the exclusion zone is balanced and does not introduce bias favoring one model over another. As such, we believe the reported results accurately represent the underlying phenomena and offer a robust assessment of model performance, mitigating the inherent measurement noise from the cuff-based reference.

For full transparency, both sets of results are reported—with and without the exclusion zone. The results without the exclusion zone, as well as analyses with alternative thresholds for high BP, are provided in the Supplementary Materials.

## Conclusions

This study represents a significant advancement in the search for BP markers in PPG signals and their applications in digital health. Our results demonstrate that PPG signals contain information that can be used to estimate BP values and distinguish between high and normal BP levels, eliminating the need for any external calibration adjustments (with and without a ±8 mmHg exclusion zone to account for cuff measurement uncertainty). This pivotal finding represents the advent of a new era in BP monitoring, utilizing the optical sensors already present in a wide range of clinical and wearable devices. By unlocking this capability, our research lays the groundwork for the widespread adoption of non-invasive, continuous BP measurement technologies, promising to significantly enhance hypertension management and healthcare delivery on a global scale.

## Data Availability

The datasets presented in this article are not readily available, but the data that support the findings of this study can be made available by Aktiia SA. However, restrictions apply to the availability of these data, which were used under license for the current study, and so are not publicly available. Therefore, availability of such data may be limited, and any access, if permissible, is subject not only to a justifiable request to be approved by Aktiia, but also to specific terms and conditions to be agreed. Requests to access the datasets should be directed to publication@aktiia.com.
