# Supplementary material for: The quest for blood pressure markers in photoplethysmography and its applications in digital health
Source: Front Digit Health. 2025 Apr 30;7:1518322. doi: 10.3389/fdgth.2025.1518322 (PMC12075524; doi:10.3389/fdgth.2025.1518322)
Supplement: Supplementary file 1 [file Datasheet1.docx]

**The Quest for Blood Pressure Markers in Photoplethysmography and its Applications in Digital Health**

Josep Sola^1^*, Andreu Arderiu^1^, Tiago P Almeida^1^*, Sibylle Fallet^1^, Sasan Yazdani^1^, Serj Haddad^1^,

David Perruchoud^1^, Olivier Grossenbacher^1^, Jay Shah^1^

^1^Aktiia SA, Neuchâtel, Switzerland.

**Supplementary Materials**

***Address for correspondence:**

Dr Josep Sola

Dr Tiago Paggi de Almeida

Rue du Bassin 8a, 2000 Neuchâtel, Switzerland

Email: publication@aktiia.com**Methods**

## ML models for BP estimation

Figure 1 illustrates the six XGBoost models created in this study for BP estimation.

##
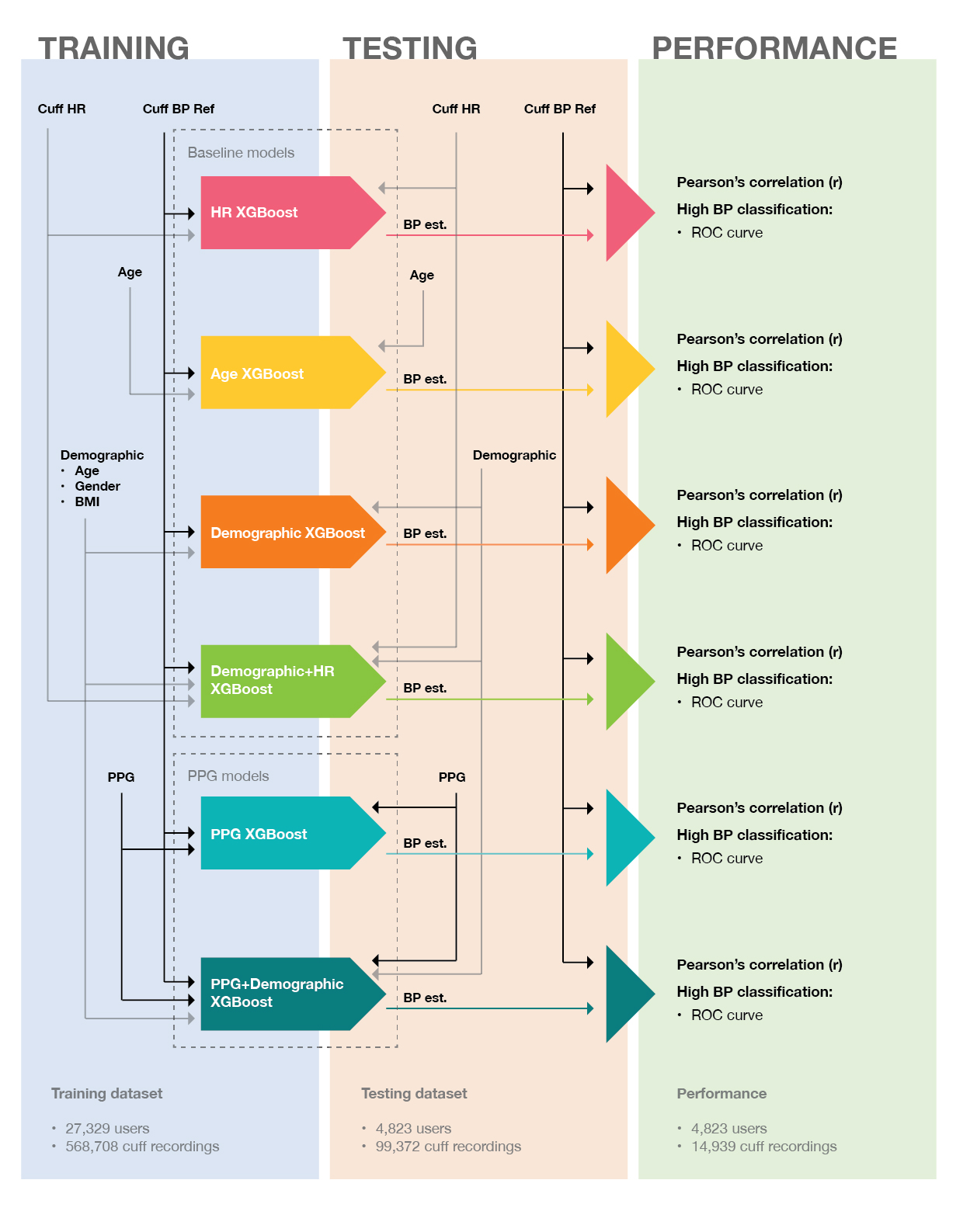


**Figure S1. Graphical representation of the six XGBoost models created in this study for BP estimation.** A total of 668,080 simultaneous cuff blood pressure (BP) and photoplethysmographic (PPG) readings (32,152 users) were used in the analysis. Data were split into 70% for training (22,506 users, 468,698 readings), 15% for validation (4,823 users, 100,010 readings), and 15% for testing (4,823 users, 99,372 readings). The performance analysis included only readings from the first day of data collection, with 14,939 cuff readings (15.0% of the whole testing set). Testing data were exclusively used to ensure no overlap with training or validation. Each model was trained with a specific input set. Four baseline models utilized heart rate (HR), Age, Demography ‘DEM’ (age, gender, height, weight), and DEM+HR as predictors, treating all physiological data except HR as constant, matched and repeated for each cuff reading across both the training and testing datasets. Two PPG-based models were evaluated, one using only PPG data and another combining PPG+DEM data. Each PPG-based model matched 30-s PPG segments with corresponding cuff readings for both training and testing. Models’ performances were analysed via correlation and ROC curves for high BP classification, with high BP defined as cuff SBP ≥ 140 mmHg and DBP ≥ 90 mmHg (separately).

## Duration of participation per participant

The distribution of distribution of study duration per participant is shown in Figure S2. Approximately 25% of the subjects (8,210 individuals out of 32,152) have data collected in one day. This is still representative as PPG signals have been collected simultaneously with cuff BP in all cases, and the analyses for the testing included only readings from the first day of each user’s monitoring period.


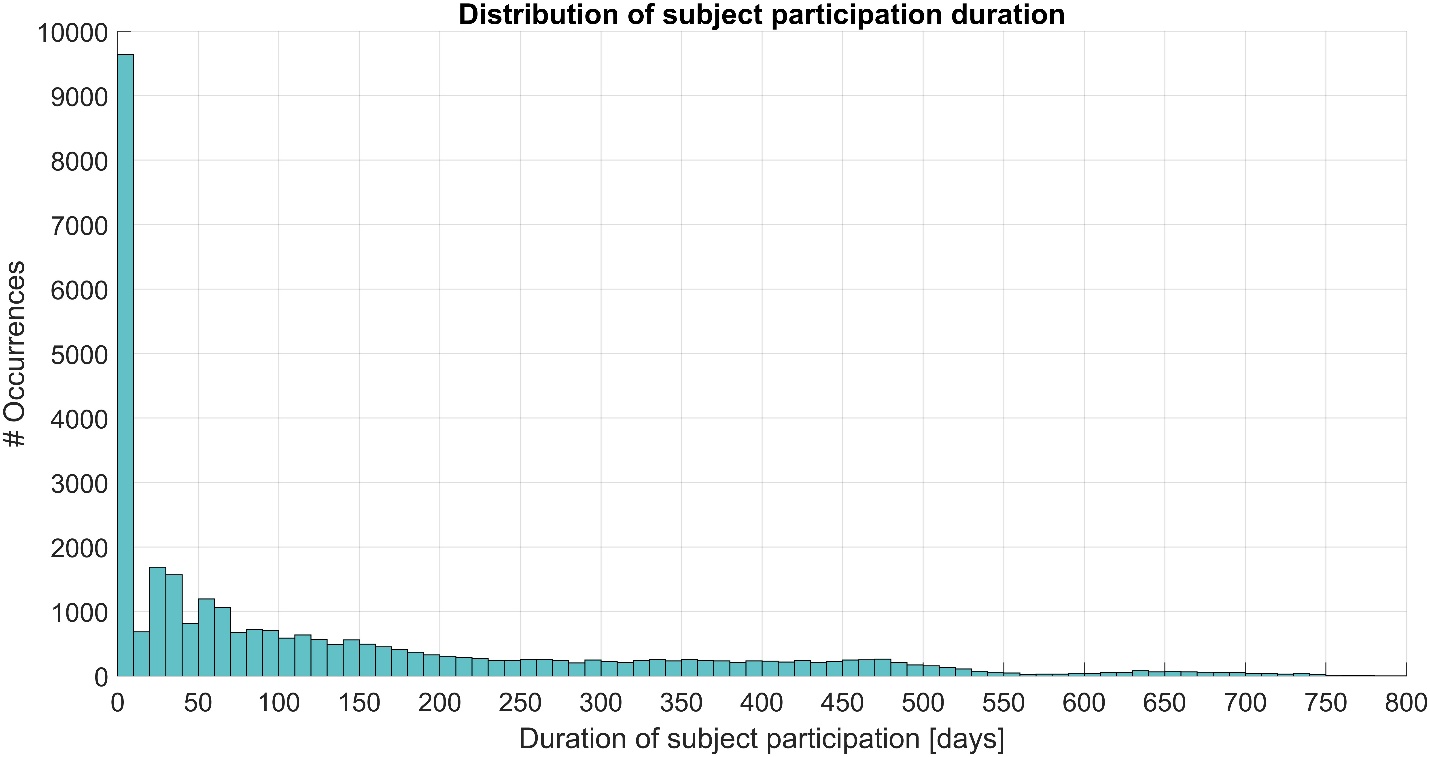


**Figure S2. distribution of study duration per participant.**

## ML models for BP estimation

The XGBoost algorithm was employed in the six models implemented in the present work (3). XGBoost is a suitable choice for building baseline models in this work due to its ability to handle structured data, capture nonlinear relationships, and efficiently process large datasets. It provides a robust framework for exploring the influence of physiological data on BP estimation accuracy. Table S1 shows the details about the parameter configuration used in the present work.

**Table S1.** Hyperparameters used for training the XGBoost model. Hyperparameters not detailed here were set to the default ones provided by the xgboost 2.0.2 python package.

| Hyperparameters | Values | Description |
| --- | --- | --- |
| Objective | reg:squarederror | Mean square error loss function for regression problems |
| tree_method | hist | Histogram tree method |
| early_stopping_rounds | 20 | Validation metric needs to improve at least once in every early_stopping_rounds round(s) to continue training. |
| learning_rate | 0.01 | Boosting learning rate |
| n_estimators | 10,000 | Number of trees to be generated |

Results

Figures S3A to S3H highlight the correlation between estimated BP with the different models and cuff BP values across the different demographic groups. These results confirm the superior BP estimation accuracy of PPG-based models across all examined demographics.

**
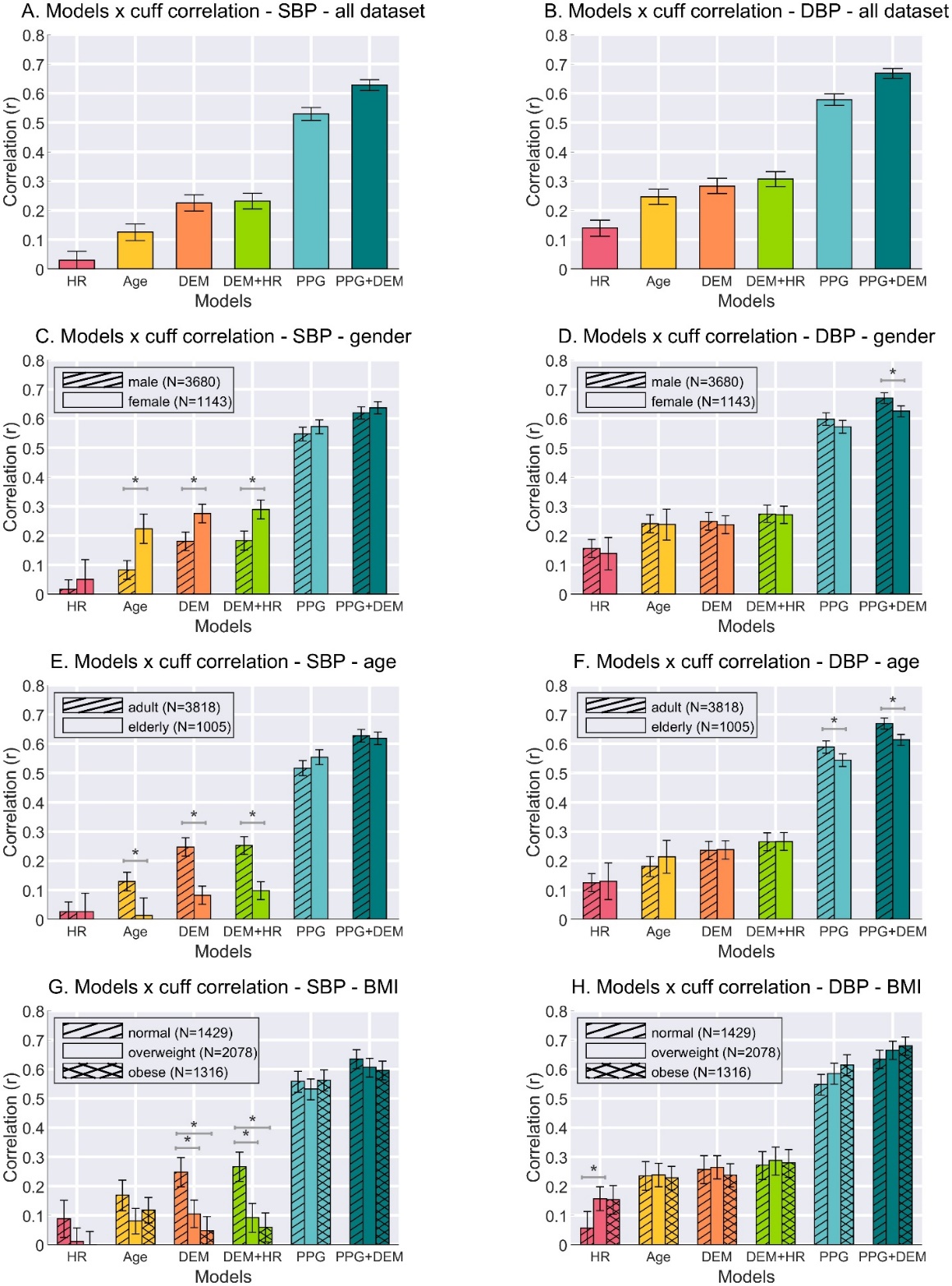
**

**Figure S3. Caption next page.**

**Figure S3 caption.** Pearson’s correlation between BP estimates from each baseline model (HR, Age, DEM, DEM+HR) and PPG-based model (PPG, PPG+DEM) versus cuff BP values across the entire testing dataset for SBP (A) and DBP (B), categorized by gender for SBP (C) and DBP (D), by age for SBP (E) and DBP (F), and by BMI classification for SBP (G) and DBP (H). Each category-specific figure includes the number of users in its respective legend. Error bars represent 95% confidence interval (CI) of the mean correlation from bootstrapping the samples (10,000 replications). Non-overlapping error bars indicate significant differences among groups (*).

A visual representation of the ROC curves and the metrics used to assess the performance of high BP classification without the ±8 mmHg exclusion zone for each model is shown in Figure S4, considering only one criterion for high SBP (≥140 mmHg) and DBP (≥90 mmHg).


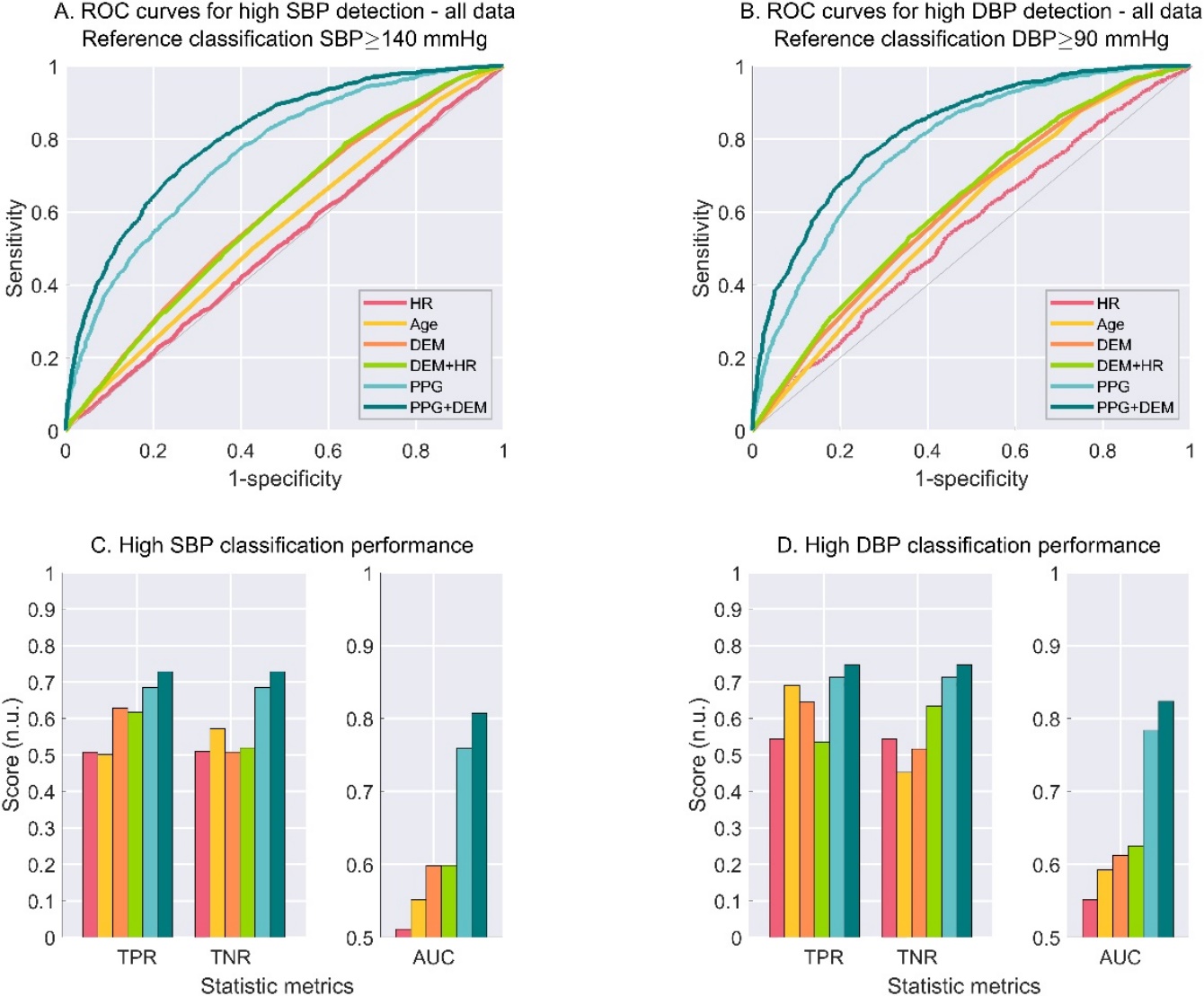


**Figure S4.** Performance for high BP classification without the ±8 mmHg exclusion zone for each model considering the criterion for high BP set at cuff SBP ≥ 140 mmHg (left-hand side), and cuff DBP ≥ 90 mmHg (right-hand side). A. ROC curves for high SBP classification. B. ROC curves for high DBP classification. C. Classification performance metrics as True Positive Rate (TPR), True Negative Rate (TNR), and Area Under the Curve (AUC) for high SBP. C. Classification performance metrics for high DBP.

The classification performance without the ±8 mmHg exclusion zone for the different criteria for high SBP and high DBP are detailed on Tables S2 and S3, respectively. PPG-based models demonstrated superior BP classification capabilities compared to baseline models for all metrics and all criteria, particularly when DEM data was incorporated.

**Table S2.** High SBP classification performance for each model and different criteria for high SBP without exclusion zone of ±8 mmHg. The bold values referring to threshold SBP ≥ 140 mmHg are illustrated in Figure S2.

| Metric | Model | Threshold for high SBP, mmHg | | | | |
| --- | --- | --- | --- | --- | --- | --- |
|  |  | >120 | >130 | **>140** | >160 | >180 |
| TPR |  |  |  |  |  |  |
|  | HR | 0.496 | 0.497 | **0.508** | 0.501 | 0.508 |
|  | Age | 0.460 | 0.479 | **0.501** | 0.558 | 0.623 |
|  | DEM | 0.566 | 0.598 | **0.628** | 0.561 | 0.574 |
|  | DEM+HR | 0.569 | 0.588 | **0.617** | 0.553 | 0.557 |
|  | PPG | 0.654 | 0.669 | **0.685** | 0.744 | 0.787 |
|  | PPG+DEM | 0.728 | 0.714 | **0.729** | 0.772 | 0.820 |
| TNR |  |  |  |  |  |  |
|  | HR | 0.504 | 0.506 | **0.510** | 0.504 | 0.513 |
|  | Age | 0.585 | 0.585 | **0.571** | 0.556 | 0.550 |
|  | DEM | 0.601 | 0.550 | **0.508** | 0.617 | 0.606 |
|  | DEM+HR | 0.605 | 0.564 | **0.519** | 0.612 | 0.602 |
|  | PPG | 0.655 | 0.669 | **0.685** | 0.744 | 0.788 |
|  | PPG+DEM | 0.728 | 0.714 | **0.729** | 0.772 | 0.820 |
| AUC |  |  |  |  |  |  |
|  | HR | 0.501 | 0.499 | **0.511** | 0.518 | 0.518 |
|  | Age | 0.542 | 0.547 | **0.552** | 0.576 | 0.612 |
|  | DEM | 0.624 | 0.600 | **0.598** | 0.620 | 0.636 |
|  | DEM+HR | 0.626 | 0.604 | **0.599** | 0.623 | 0.628 |
|  | PPG | 0.724 | 0.730 | **0.759** | 0.830 | 0.895 |
|  | PPG+DEM | 0.804 | 0.792 | **0.807** | 0.857 | 0.894 |

**Table S3.** High DBP classification performance for each model and different criteria for high DBP without exclusion zone of ±8 mmHg. The bold values referring to threshold DBP ≥ 90 mmHg are illustrated in Figure S2.

| Metric | Model | Threshold for high DBP, mmHg | | | | |
| --- | --- | --- | --- | --- | --- | --- |
|  |  | >80 | >85 | **>90** | >100 | >110 |
| TPR |  |  |  |  |  |  |
|  | HR | 0.555 | 0.553 | **0.544** | 0.568 | 0.609 |
|  | Age | 0.654 | 0.662 | **0.691** | 0.704 | 0.685 |
|  | DEM | 0.602 | 0.623 | **0.645** | 0.479 | 0.587 |
|  | DEM+HR | 0.613 | 0.631 | **0.536** | 0.557 | 0.522 |
|  | PPG | 0.707 | 0.701 | **0.713** | 0.751 | 0.837 |
|  | PPG+DEM | 0.731 | 0.737 | **0.746** | 0.784 | 0.837 |
| TNR |  |  |  |  |  |  |
|  | HR | 0.555 | 0.552 | **0.544** | 0.565 | 0.606 |
|  | Age | 0.521 | 0.474 | **0.452** | 0.425 | 0.418 |
|  | DEM | 0.593 | 0.549 | **0.517** | 0.648 | 0.477 |
|  | DEM+HR | 0.612 | 0.557 | **0.634** | 0.602 | 0.593 |
|  | PPG | 0.707 | 0.701 | **0.713** | 0.751 | 0.834 |
|  | PPG+DEM | 0.731 | 0.737 | **0.747** | 0.784 | 0.836 |
| AUC |  |  |  |  |  |  |
|  | HR | 0.567 | 0.567 | **0.552** | 0.579 | 0.631 |
|  | Age | 0.618 | 0.591 | **0.592** | 0.583 | 0.559 |
|  | DEM | 0.637 | 0.619 | **0.612** | 0.611 | 0.578 |
|  | DEM+HR | 0.655 | 0.635 | **0.625** | 0.628 | 0.613 |
|  | PPG | 0.772 | 0.773 | **0.784** | 0.833 | 0.902 |
|  | PPG+DEM | 0.819 | 0.815 | **0.823** | 0.867 | 0.919 |

References

1. Sola J, Vybornova A, Fallet S, Polychronopoulou E, Wurzner-Ghajarzadeh A, Wuerzner G. Validation of the optical Aktiia bracelet in different body positions for the persistent monitoring of blood pressure. Sci Rep. 2021 Dec;11(1):20644.

2. Vybornova A, Polychronopoulou E, Wurzner-Ghajarzadeh A, Fallet S, Sola J, Wuerzner G. Blood pressure from the optical Aktiia Bracelet: a 1-month validation study using an extended ISO81060-2 protocol adapted for a cuffless wrist device. Blood Pressure Monitoring. 2021 Aug;26(4):305–11.

3. Chen T, Guestrin C. XGBoost: A Scalable Tree Boosting System. In: Proceedings of the 22nd ACM SIGKDD International Conference on Knowledge Discovery and Data Mining. San Francisco California USA: ACM; 2016. p. 785–94.
